# Postural changes in optic nerve and optic nerve sheath diameters in postural orthostatic tachycardia syndrome and spontaneous intracranial hypotension: A cohort study

**DOI:** 10.1371/journal.pone.0223484

**Published:** 2019-10-09

**Authors:** Debora Cipriani, Belén Rodriguez, Levin Häni, Raya Zimmermann, Jens Fichtner, Christian T. Ulrich, Andreas Raabe, Jürgen Beck, Werner J. Z‘Graggen

**Affiliations:** 1 Department of Neurosurgery, Inselspital, Bern University Hospital, Bern, Switzerland; 2 Department of Neurosurgery, Medical Center University of Freiburg, Freiburg, Germany; 3 Department of Neurology, Inselspital, Bern University Hospital, Bern, Switzerland; Universidade Federal de Juiz de Fora, BRAZIL

## Abstract

**Background:**

Postural orthostatic tachycardia syndrome is a disorder of the autonomic nervous system. Approximately 30% of patients experience orthostatic headaches. Orthostatic headaches also are a hallmark symptom in spontaneous intracranial hypotension. While the cause of orthostatic headaches in spontaneous intracranial hypotension can be linked to the cerebrospinal fluid loss at the spinal level and consecutively reduced intracranial pressure in the upright position, the cause of orthostatic headaches in postural orthostatic tachycardia syndrome still remains unknown. The present study examined orthostatic changes of intracranial pressure using dynamic ultrasound of the optic nerve and optic nerve sheath diameter in postural orthostatic tachycardia syndrome, spontaneous intracranial hypotension and healthy subjects.

**Methods:**

Data was obtained from postural orthostatic tachycardia syndrome patients with (*n* = 7) and without orthostatic headaches (*n* = 7), spontaneous intracranial hypotension patients (*n* = 5) and healthy subjects (*n* = 8). All participants underwent high-resolution transorbital ultrasound in the supine and upright position to assess optic nerve and optic nerve sheath diameter.

**Results:**

Group differences were found in percentage deviations when changing position of optic nerve sheath diameter (*p* < 0.01), but not regarding the optic nerve diameter. Pairwise comparisons indicated differences in optic nerve sheath diameter only between spontaneous intracranial hypotension and the other groups. No differences were found between postural orthostatic tachycardia syndrome patients with and without orthostatic headaches.

**Conclusion:**

This study shows that the size of the optic nerve sheath diameter dynamically decreases during orthostatic stress in spontaneous intracranial hypotension, but not in postural orthostatic tachycardia syndrome with or without orthostatic headaches, which indicates different underlying causes.

## Introduction

The Postural Orthostatic Tachycardia Syndrome (POTS) is a disorder of the autonomic nervous system characterized by symptoms of orthostatic intolerance. Orthostatic intolerance describes a condition in which patients experience symptoms only in the upright posture, which can promptly be improved by lying down [1]. The syndrome is defined in individuals aged 20 years or more by a heart rate increment (HR) of ≥ 30 beats/min (HR often ≥ 120 beats/min) within 10 minutes of head-up tilt or standing without orthostatic hypotension. In adolescents aged 12–19 years a HR increment ≥ 40 beats/min is required to reach diagnosis [2, 3]. The prevalence of POTS is estimated to be at least 170/100’000 with a female-male ratio of 5:1 [4, 5]. POTS has been classified into several subtypes [4, 6–8], with the most common subtype being the neuropathic POTS [4, 6]. This subtype is primarily characterized by a peripheral sympathetic denervation and consecutively impaired vasoconstriction in the lower limbs, leading to venous pooling upon standing. Another subtype is the hyperadrenergic POTS, which is characterized by an increase in central sympathetic drive with orthostatic plasma norepinephrine levels ≥ 600 pg/mol [6]. The most commonly reported symptoms include palpitations, tremulousness, lightheadedness, nausea, dizziness, chest pain and cognitive impairment [9]. Up to 27% of patients experience orthostatic headaches (OSH) [10]. The exact cause of OSH still remains elusive [11]. There are, however, suggestions that changes in spinal venous pressure and/or reduced cerebrospinal fluid (CSF) volume causing CSF hypotension might be underlying mechanisms [12]. Some of the position-dependent symptoms in POTS clinically overlap with symptoms of patients with spontaneous intracranial hypotension (SIH) [10]; SIH is secondary to a CSF leak at the spinal level [13–15] and affected patients typically experience OSH, neck stiffness and/or hearing symptoms [16]. The postulated mechanism of OSH in patients with SIH is that CSF loss at the spinal level leads to orthostatic brain sagging causing painful tearing at the cranial meninges. Possibly, CSF loss also increases compliance at the caudal part of the dural tube, which in turn increases brain sagging [10, 17]. High-resolution transorbital sonography of the optic nerve sheath diameter (ONSD) has been shown to sensitively detect increments [18] as well as reductions in intracranial pressure [13, 19, 20]. In SIH, it has been shown that dynamic ultrasound of the ONSD can be used as a non-invasive tool for diagnosis [13].

Based on the overlap in experienced symptoms, it could be hypothesized that OSH in POTS and OSH in SIH share similar pathophysiological mechanisms. The aim of the present study was to examine orthostatic changes of the optic nerve and optic nerve sheath diameters in POTS, SIH and healthy subjects using dynamic ultrasound as an indicator for changes of intracranial pressure.

## Methods and materials

### Participants

The study has been carried out in accordance with the Declaration of Helsinki of the World Medical Association and received approval of the local ethics committee (Kantonale Ethikkommission Bern, Switzerland). 7 patients with confirmed neuropathic POTS with OSH (5 female, mean age 25, range 18–53 years), 7 patients with neuropathic POTS without OSH (6 female, mean age 27, range 20–46 years), 5 patients with confirmed SIH (4 female, mean age 50, range 33–65 years) and 8 healthy control subjects (7 female, mean age 25, range 23–30 years) participated. Patients were asked to participate in the study in the context of their treatment at the Departments for Neurology and Neurosurgery, University Hospital Bern, Switzerland. Healthy controls were recruited through distributed flyers at the University of Bern. Both POTS and SIH patients were diagnosed within a maximum of 6 weeks prior to testing. None of the participants had to be excluded after enrollment as we were able to confirm the diagnosis in every individual. Diagnosis of neuropathic POTS was made according to published criteria [2, 3] including the following protocol: medical history, normal physical examination with the exception of possible cutaneous abnormalities because of reduced sweating, cardiovascular autonomic function testing, quantitative sudomotor axon reflex testing and/or thermoregulatory sweat test, as well as measurement of plasma norepinephrine levels and cutaneous biopsy in selected cases [2–4, 7]. Diagnosis of SIH was made according to the current guidelines of the International Headache Society [16]. These guidelines include the following criteria: (1) any headache fulfilling criterion 3; (2) absence of a procedure or trauma known to be able to cause CSF leakage; (3) headache has developed in temporal relation to occurrence of low CSF pressure or CSF leakage, or has led to its discovery; (4) not better accounted for by another International Classification of Headache Disorders 3rd edition (ICHD-3) diagnosis. Only patients with confirmed CSF leak at the spinal level were included [21]. For localization of the site of the spinal leak, a systematic diagnostic algorithm was applied [21], consisting of cranial magnetic resonance imaging (MRI), fluid-sensitive thin-slice MRI of the spinal axis, measurement of lumbar opening pressure and lumbar infusion test, spinal T1-weighted MR sequences after intrathecal gadolinium application, dynamic myelography and postmyelography spine CT imaging including sometimes also repeated films (4 h later). Every participant provided full written informed consent. Exclusion criteria included pregnancy, breastfeeding and participants aged <18 years for all participants.

### Transorbital ultrasound

**Fig 1:**
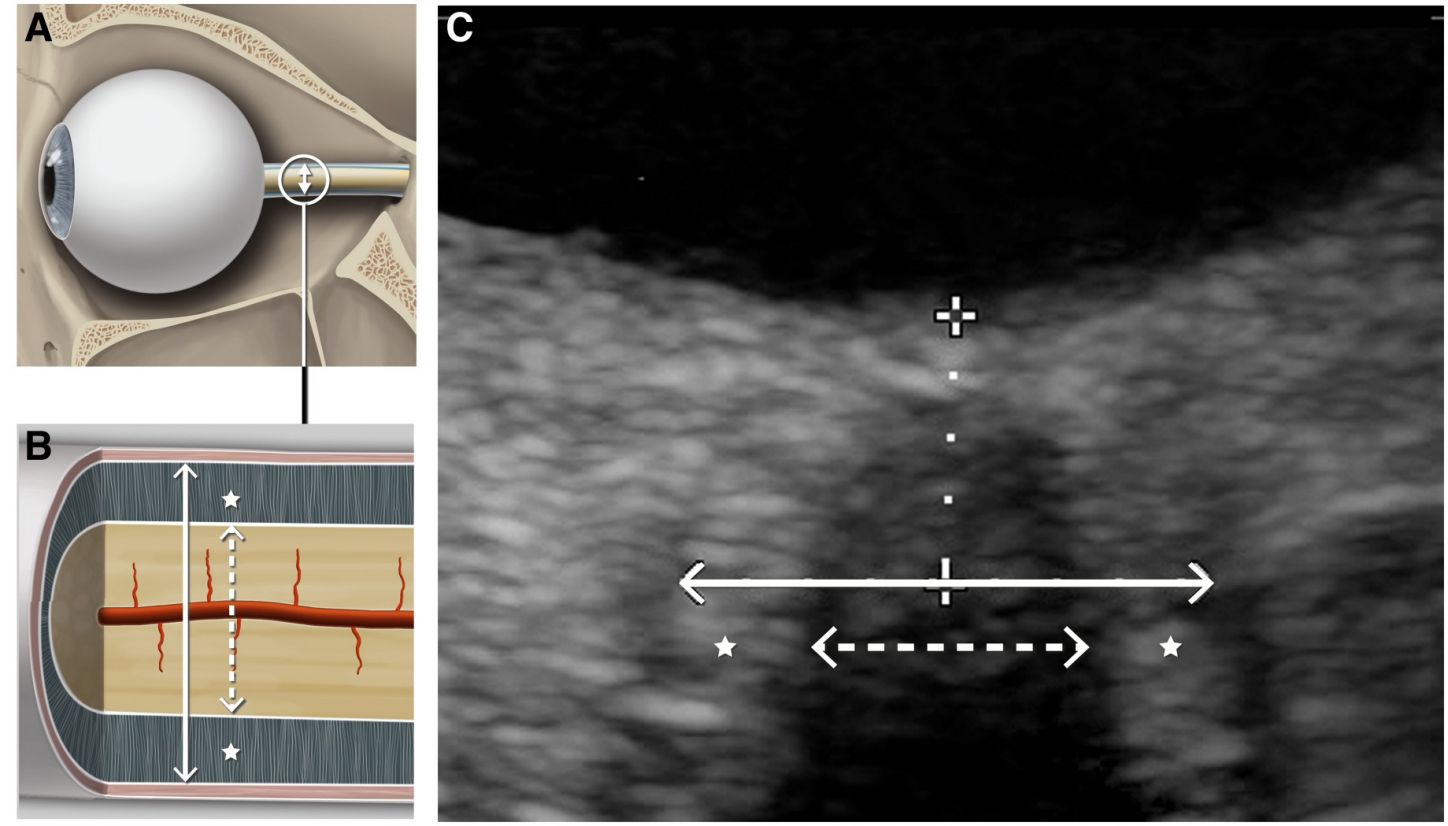
A: Illustration of the human eyeball and optic nerve. B: Magnification of the optic nerve and its compartments: The dashed arrow indicates the diameter of the optic nerve, the continuous arrow the diameter of the optic nerve sheath and the stars the perineural space. C: High-resolution transorbital ultrasound image of the optic nerve complex. The diameters of the optic nerve (dashed arrow) and optic nerve sheath (continuous arrow) were measured 3 mm behind the papilla (dotted line).

The optic nerve diameter (OND) and the ONSD of the right eye were measured with a colorduplex ultrasound machine (IU22, Philips, Amsterdam, Netherlands) using a 7–15 MHz linear array transducer. Ultrasound measurements were performed first in the supine position after a 10-minute rest period and then in the upright position. At each time of measurement, three consecutive ultrasound assessments were made by the same examiner. The probe was placed on the upper eyelid (temporal part) using a layer of gel. The OND and ONSD were measured 3 mm behind the papilla [22]. All ultrasound examinations were conducted by two trained and non-blinded examiners. The mechanical index was reduced to less than or equal to 0.23 (Fig 1).

### Statistical analysis

All statistical analyses were performed using SPSS Statistics 25.0 (IBM, Armonk, NY, USA). The Shapiro–Wilk normality test was used to test for normal distribution of all parameters. Group differences in age and gender were analysed using Kruskal-Wallis test and Chi-square test, respectively. Out of the three consecutive ultrasound measurements in the supine and upright position the largest OND and ONSD were selected and based on these values the corresponding surfaces were calculated. The diameter of the perineural space (PNSD) was calculated by first subtracting the surface of the optic nerve from the surface of the optic nerve sheath and then based on the calculated surface the diameter was derived. For each diameter the percentage change between the supine and upright position was calculated. Absolute diameters in the supine and upright position were analyzed using 2x4 analyses of variance (ANOVA) for repeated measures with Bonferroni correction for multiple comparisons. The factors were i) position (supine; upright) and ii) group (POTS with OSH; POTS without OSH; SIH; control subjects). Percentage changes between the supine and upright position were statistically compared using one-way ANOVAs with Bonferroni correction for multiple comparisons. Group data are reported as mean ± S.E.M. A two-tailed *P* value ≤ 0.05 was defined as statistically significant. To determine the sample size, an *a priori* power analysis was performed based on previous data concerning ONSD changes in SIH patients [13, 23] using G*Power [24]. The analysis indicated that a total sample of 24 people would be needed to detect medium effects (Cohen’s *d* = 0.533) with an 80% power using a mixed model ANOVA for repeated measures with alpha set at 0.05 with four experimental groups and two measurements each.

## Results

### Participant characteristics

Demographics of all participants are summarized in Table 1. Overall, POTS patients and control subjects were younger than SIH patients (*p* = 0.006).

**Table 1:** Participant characteristics.

	POTS + OSH	POTS—OSH	SIH	Control	*P* value
*N*	7	7	5	8	
Age *Mean (Range)*	25 (18–53)	27 (20–46)	50 (33–65)	25 (23–30)	0.006*
Sex *f (%)*	5 (71%)	6 (86%)	4 (80%)	7 (88%)	0.868

*Note*. Age is reported in years.

*P ≤ 0.05

POTS, postural orthostatic tachycardia syndrome; OSH, orthostatic headache; SIH, spontaneous intracranial hypotension; f, female.

### Transorbital ultrasound

**Fig 2:**
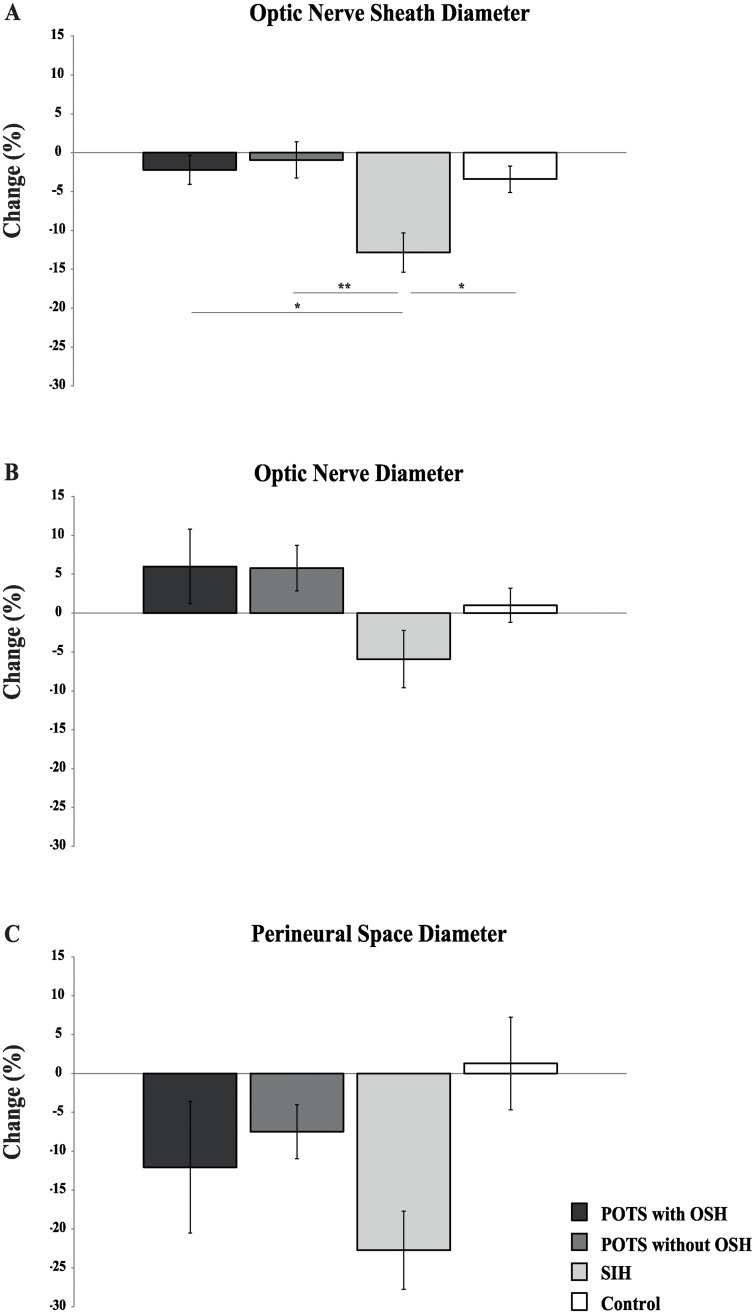
Bar graphs showing diameter changes (supine to standing) of A) the optic nerve sheath, B) optic nerve and C) perineural space. Values are given as means ± S.E.M. * *p* ≤ 0.05; ** *p* ≤ 0.01. POTS, postural orthostatic tachycardia syndrome; OSH, orthostatic headache; SIH, spontaneous intracranial hypotension.

Group mean values of ONSD, OND and PNSD for the supine and upright position are shown in Table 2. ONSD showed a main effect of position (F(3, 23) = 22.18, *p* < 0.001) and a significant interaction between position and group (F(3, 23) = 6.41, *p* = 0.003). OND showed neither main effects nor an interaction. PNSD showed a main effect of position (F(3, 23) = 14.68, *p* = 0.001) and a significant interaction between both factors (F(3, 23) = 3.03, *p* = 0.050). Percentage changes of ONSD, OND and PNSD between the supine and upright position are illustrated in Fig 2. Analysis of ONSD showed significant group differences (F(3, 23) = 5.209, *p* = 0.007). Pairwise comparisons indicated differences between POTS patients with OSH and SIH patients (*p* = 0.020), POTS patients without OSH and SIH patients (*p* = 0.008) and SIH patients and healthy subjects (*p* = 0.040). Group differences were not significant regarding OND (F(3, 23) = 2.473, *p* = 0.087) and PNSD (F(3, 23) = 2.193, *p* = 0.116).

**Table 2:** Transorbital ultrasound data.

	POTS + OSH *(n = 7)*	POTS—OSH *(n = 7)*	SIH *(n = 5)*	Control *(n = 8)*	*P* value
**Optic nerve sheath diameter**					0.003**
Supine	4.8 ± 0.1	4.5 ± 0.2	5.3 ± 0.1	4.8 ± 0.2	
Upright	4.8 ± 0.2	4.5 ± 0.2	4.6 ± 0.1	4.6 ± 0.2	
**Optic nerve diameter**					0.080
Supine	3.7 ± 0.3	3.1 ± 0.2	3.5 ± 0.2	3.8 ± 0.2	
Upright	3.8 ± 0.2	3.3 ± 0.2	3.4 ± 0.2	3.6 ± 0.2	
**Perineural space diameter**					0.050*
Supine	2.5 ± 0.1	2.6 ± 0.2	3.1 ± 0.1	2.2 ± 0.1	
Upright	2.1 ± 0.2	2.4 ± 0.1	2.4 ± 0.2	2.2 ± 0.1	

*Note*. Values are reported in mm. Data are reported as mean ± S.E.M. *P* values refer to the interactions of group and position, derived from analyses of variance for repeated measures.

*P ≤ 0.05;

**P ≤ 0.01

POTS, postural orthostatic tachycardia syndrome; OSH, orthostatic headache; SIH, spontaneous intracranial hypotension

## Discussion

OSH is the leading symptom of SIH. Up to 27% of patients with POTS also experience OSH [10] as a prominent symptom of orthostatic intolerance. In SIH, OSH is attributed to orthostatic brain sagging causing painful stretching of the cranial meninges because of CSF loss at the spinal level [17]. The spinal fluid loss results in a reduction of the cranial CSF volume and is probably paralleled by a drop in intracranial pressure. By now, the cause of OSH in POTS is still unknown. Due to the clinical similarities of OSH in SIH and POTS, the present study examined the different compartments of the optic nerve (OND, PNSD and ONSD) by performing consecutive high-resolution transorbital ultrasound assessments both in the supine and upright position in POTS patients with and without OSH, SIH patients and healthy controls. Whereas a significant position-dependent reduction of the ONSD was found in SIH patients, no positional changes were evident in both groups of POTS patients and healthy controls.

Transorbital ultrasound of the ONSD has gained high diagnostic value for non-invasively assessing changes of intracranial pressure, both increments and reductions [13, 18–20]. It was further shown that transorbital ultrasound of the OND can depict optic nerve atrophy in Multiple Sclerosis [25]. Recently, Lochner et al. (2016) reported that this technique is a feasible method to assess both ONSD and OND with a high intra- and interobserver reliability [26]. Consequently, the use of the technique has been studied in different diseases, e.g. idiopathic normal-pressure hydrocephalus, idiopathic intracranial hypertension and SIH. In idiopathic normal-pressure hydrocephalus, sonography of ONSD was shown to be a reliable diagnostic supplement to spinal tap tests [27]. Furthermore, ONSD has consistently been found to be bigger in patients with idiopathic intracranial hypertension than in controls [28], whereas transorbital ultrasound of OND did not reveal such a difference [29]. In SIH, it has been shown that dynamic ultrasound of the ONSD can be used as a non-invasive tool for diagnosis [13] and follow-up after surgical treatment of the CSF leak [23].

The results of transorbital ultrasound of the optic nerve compartments derived from the healthy control group in the present study match published standard values [30, 31] and the values derived from SIH patients correspond with similar prior studies [13, 23]. In contrast to our hypothesis the ultrasound data obtained from POTS patients do not point towards common underlying mechanisms of OSH between SIH and POTS patients. While the size of the ONSD dynamically decreases during orthostatic stress in SIH patients, it remains stable in POTS patients (and healthy controls). Furthermore, no differences were found between POTS patients with and without OSH and additionally also controls. Hence, POTS patients with and without OSH seem to exhibit an intact positional regulation of intracranial pressure and therefore, OSH in POTS is probably not attributed to a reduction of intracranial pressure as in SIH. Additionally, no differences of OND were found in any group.

The purpose of the present study was to examine if patients with POTS and SIH possibly share the same cause of OSH. In showing that POTS patients neither with nor without OSH do not present with orthostatic changes of ONSD our results are not in support of this hypothesis. Therefore, it has to be assumed that OSH in POTS cannot be attributed to orthostatic changes of intracranial pressure. Due to the overlap of clinical symptoms between SIH and POTS the International Headache Society states in their current version of diagnostic criteria for SIH that in patients with typical orthostatic headache and no apparent cause POTS should be excluded before any treatment attempt with lumbar epidural blood patch is made [16]. A recently published retrospective study showed that screening autonomic function testing is not appropriate to differentiate between POTS and SIH [10]. Based on the results of our study and earlier reports [13], we propose that dynamic high-resolution transorbital sonography of the ONSD could be used as a non-invasive, fast and easy to perform screening tool for differential diagnosis of SIH and POTS with OSH, which may allow the clinician to omit an extensive and costly evaluation for a CSF leak at the spinal level.

### Limitations

The findings of the present study should be viewed in light of some limitations. First, the study was conducted with a small sample size, especially regarding the SIH group. It was therefore not feasible to control for a potential confounding effect of age. However, it has to be taken into account that the two pathologies in question present with different age ranges per se [32, 33] and since the results of our SIH cohort and control group are well in line with earlier reports, we consider the risk for a possible bias as negligible. Second, high-resolution transorbital ultrasound of the optic nerve compartments was shown to correlate with measurements of intracranial pressure and dynamic ultrasound with changes of intracranial pressure, but does not allow to measure absolute values of intracranial pressure.

### Conclusion

In conclusion, this study shows that the size of the ONSD dynamically decreases during orthostatic stress in SIH patients, but not in POTS patients with or without OSH. This finding is indicative of different underlying causes of OSH in SIH and POTS.

## Supporting information

S1 Dataset Data derived from all optic nerve ultrasound examinations. (XLSX)
